# Thyrotoxic Cardiomyopathy: A Rare Case of Thyroid Storm Presenting as De Novo Heart Failure

**DOI:** 10.7759/cureus.57924

**Published:** 2024-04-09

**Authors:** Stefanie Clinton Fotso Simo, Kai Shiang Lin, Inna Bukharovich

**Affiliations:** 1 Internal Medicine, State University of New York Downstate Medical Center, Brooklyn, USA; 2 Cardiology, Kings County Hospital Center, Brooklyn, USA

**Keywords:** cardiomyopathy, dilated cardiomyopathy, heart failure, thyroid storm, thyrotoxic cardiomyopathy

## Abstract

Thyroid storm and heart failure represent formidable challenges in clinical practice. Their coexistence, however, poses an even greater threat to the patient's well-being. To facilitate early recognition and appropriate management, an understanding of the complex interplay between these two conditions is crucial. Through comprehensive assessment, vigilant monitoring, and prompt intervention, outcomes can be improved and the morbidity and mortality mitigated. We present a rare case of a 40-year-old male who presented with severe de novo heart failure and a concurrent thyroid storm. Despite an initial left ventricular systolic ejection fraction of 20% and evidence of global dilatation and hypokinesis on echocardiography, appropriate management resulted in improved clinical status and ultimately recovery of cardiac function.

## Introduction

Heart failure and thyroid storm are relatively rare complications of thyrotoxicosis. A combination of both clinical entities, thyrotoxic cardiomyopathy (TCM) is even rarer and can present with severe impairment of left ventricular (LV) function with possible cardiogenic shock. TCM is most commonly associated with Graves' disease, the most common cause of hyperthyroidism, and is thought to develop in approximately 1% of patients with thyrotoxicosis, although its exact prevalence and incidence remain elusive due to the scarcity of large-scale epidemiological studies [1,2]. Similarly, the incidence of thyroid storm is also rare, with one study reporting its incidence to be 0.57-0.76 cases per 100,000 US persons per year and 4.8-5.6 cases per 100,000 hospitalized patients per year [3]. Despite the rarity of TCM and thyroid storm, it is crucial to remain cognizant of both clinical entities due to their high morbidity and mortality, as up to 75% of hospitalized patients can expire, particularly when TCM presents in the setting of thyroid storm [4]. Early recognition of TCM can also be beneficial given the potential for reversibility when promptly treated, first postulated in the 1980s [5]. In this report, we present a rare case of TCM in the setting of a thyroid storm and highlight the clinical presentation, imaging findings, diagnostic approach, and management of this unique clinical scenario.

## Case presentation

A 40-year-old male with no known past medical history presents to the emergency department with a one-week history of new-onset lower extremity swelling that has progressed to his lower abdomen and scrotum. The lower extremity swelling is bilateral and has increasingly affected his ability to ambulate. It is not associated with pain, paresthesias, rashes, or other skin changes. A review of systems was remarkable for intermittent palpitations, mild abdominal bloating, and an unintentional weight loss of approximately 30 pounds over the last year but negative for chest pain, cough, orthopnea, paroxysmal nocturnal dyspnea, shortness of breath, abdominal distension, eye puffiness, or appetite changes. He also denies lightheadedness, loss of consciousness, unilateral limb weakness, anxiety, tremors, insomnia, and heat intolerance, although he did report the use of herbal and tonic supplements for his abdominal bloating two weeks prior to presentation. He denies any other constitutional symptoms and considered himself to be healthy prior to the onset of the aforementioned symptoms. Notably, he does not see a doctor regularly.

His smoking history is less than 1 pack-year, and he admits to occasional alcohol use but not recreational or illicit drugs. He also denies recent travel or illnesses and has no notable past surgical history or known drug allergies. His family history is remarkable only for hypertension in his father, and he lives at home with his girlfriend. His occupation involves floor installation. 

**Figure 1:**
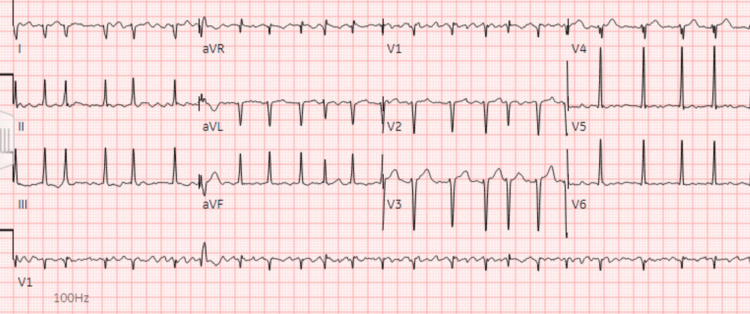
EKG on admission showing atrial fibrillation with rapid ventricular response with premature ventricular or aberrantly conducted complexes. EKG: electrocardiogram

**Figure 2:**
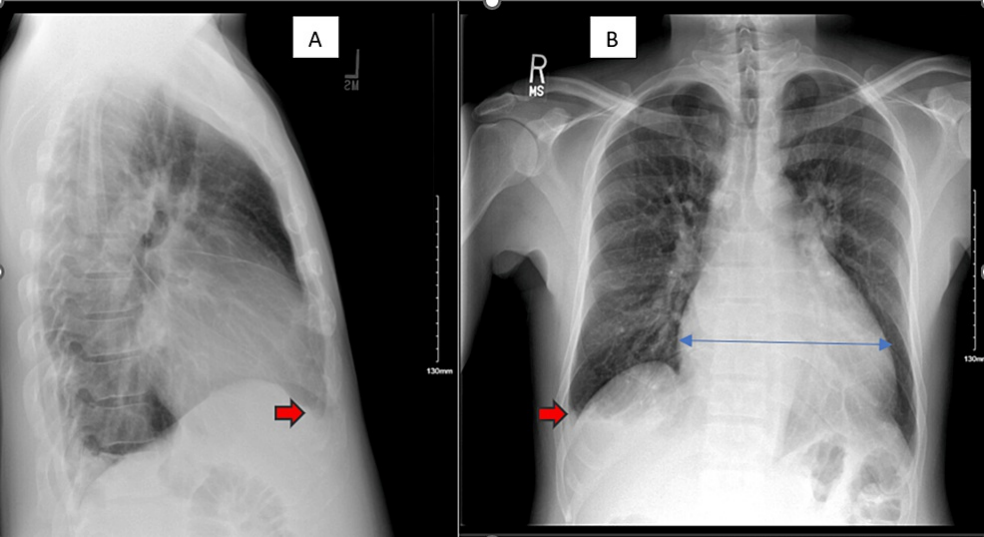
Chest X-ray on admission showing significant cardiomegaly (blue arrow) and right-sided pleural effusion (red arrows) with atelectasis and/or infiltrate. Image A is a lateral view; image B is a posterior-anterior view.

On presentation, his vital signs were notable for a blood pressure of 105/92 millimeters mmHg, heart rate of 112-154 beats per minute, temperature of 37.1°C, respiratory rate of 19 per minute, and oxygen saturation at 98% in room air. Physical exam revealed a non-toxic appearance, irregularly irregular tachycardia, and 2+ pitting edema in the bilateral lower extremities extending to the scrotum without any tenderness, erythema, or other signs of infection. No jugular venous distension, hepatojugular reflux, or ophthalmopathy was appreciated. Respiratory effort was normal, and the lungs were clear to auscultation bilaterally. His abdomen was slightly distended but soft and non-tender. Laboratory workup revealed a hemoglobin of 13.1 g/dL, a white blood cell count of 5.34×109/L, an N-terminal prohormone of brain natriuretic peptide (NT-proBNP) of 1,240 pg/mL, and a troponin T level within normal limits. An electrocardiogram (EKG) showed atrial fibrillation (AF) with rapid ventricular response (RVR) at a rate of 139 (Figure 1), and a chest X-ray showed significant cardiomegaly and right-sided pleural effusion with atelectasis and/or infiltrate (Figure 2). Due to concern for impending decompensation, the patient was admitted to the telemetry unit for AF with RVR and new-onset heart failure.

**Figure 3:**
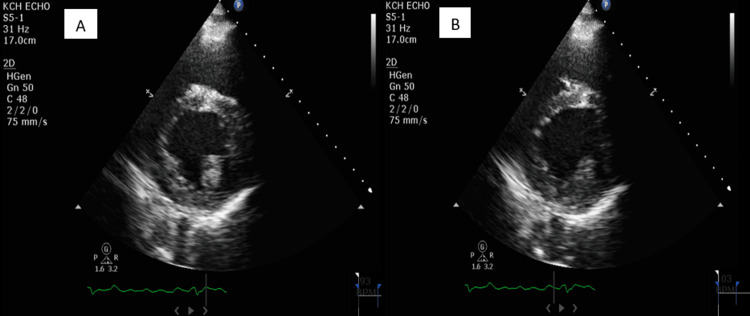
Transthoracic echocardiogram in the parasternal short-axis view on admission. Image A shows the LV in systole. Image B shows the LV in diastole. The ejection fraction is approximately 20%. LV: left ventricle

**Figure 4:**
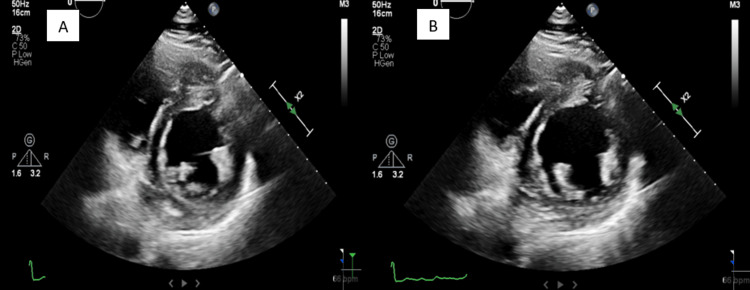
Transthoracic echocardiogram in the parasternal short-axis view four months after admission. Image A shows the LV in systole. Image B shows the LV in diastole. The ejection fraction has improved significantly from approximately 20% to approximately 40%. LV: left ventricle

Once admitted, the patient was started on rate control agents and diuresis. Serial troponins were consistently within normal limits, but transthoracic echocardiography (TTE) revealed an ejection fraction of 20%, severe biatrial dilation, mild LV dilation, and severe mitral regurgitation (Figure 3). Further laboratory workup revealed a thyroid-stimulating hormone (TSH) level of <0.010 (normal range: 0.270-4.200 uIU/mL), a free T4 level of 6.06 (normal range: 0.93-1.70 ng/dL), a thyroid-stimulating immunoglobulin (TSIG) level of 9.51 IU/L (normal range: 0.00-0.55 IU/L), a thyrotropin receptor (TSHR) antibodies level of 14 IU/L (normal range: 0.00-1.75 IU/L), and a total T3 level of 258 ng/dL (normal range: 80-200 ng/dL) (Table 1). The Burch-Wartofsky Point Scale (BWPS) score was 55, suggesting thyroid storm in the setting of Graves' disease, and methimazole was started. Once the patient was clinically stabilized, he was discharged on antithyroid drug therapy and guideline-directed medical therapy for Graves' disease and heart failure with reduced ejection fraction, respectively. A repeat TTE done four months later at an outpatient follow-up was remarkable for an ejection fraction of 40%, moderate biatrial dilation, mild LV dilation, and moderate mitral regurgitation (Figure 4). A repeat thyroid function test done two months post-discharge was within normal limits. The patient remained in AF.

**Table 1:** Laboratory results on admission. TSH: thyroid-stimulating hormone

Test name	Result	Reference range	Units
N-terminal pro-B-type natriuretic peptide	1240 (high)	<125	pg/ml
TSH	<0.010 (low)	0.270-4.200	uIU/mL
Triiodothyronine	258 (high)	80-200	ng/dL
Free thyroxine	6.06 (high)	0.93-1.70	ng/dL
Thyroid-stimulating immunoglobulin	9.51 (high)	0.00-0.55	IU/L
TSH receptor antibody	14 (high)	0.00-1.75	IU/L

## Discussion

Thyrotoxicosis is a condition in which inappropriately high levels of circulating thyroid hormone result in a heterogeneous presentation ranging from the asymptomatic state to the hypermetabolic, life-threatening thyroid storm. It is usually caused by hyperthyroidism, a form of thyrotoxicosis secondary to excessive endogenous thyroid hormone production, but can also be caused by exogenous etiologies such as factitious hyperthyroidism and excessive levothyroxine replacement therapy. A specific subset of patients can present with an exaggerated form known as thyroid storm, a rare medical emergency with an estimated prevalence of 16% in patients hospitalized with thyrotoxicosis [3]. For reasons that are still unclear, the level of thyroid hormone elevation correlates poorly with the incidence of thyroid storm, and the exact mechanism by which thyroid storm develops remains to be elucidated, despite prevailing theories such as rapid increases in thyroid hormone levels, sudden cessation of antithyroid drug therapy, increased sensitivity to catecholamines, and heightened responses to thyroid hormone at the cellular level [6-8]. Irrespective of the exact mechanism, thyroid storm appears to be frequently preceded by an acute precipitating event such as infection, an acute iodine load, or thyroid surgery, although it can also be seen with longstanding, untreated hyperthyroidism.

Symptoms of thyroid storm resemble those of hyperthyroidism, such as palpitations, weight loss, and tremors, but with significantly greater severity. In addition, thyroid storm can present with cardiovascular manifestations that can be both devastating and life-threatening [9]. Reduced exercise tolerance is the most common cardiovascular manifestation, whereas other manifestations such as AF, pulmonary hypertension, and angina pectoris are less common [10]. Rarely, with a prevalence of 6% and 1%, respectively, thyroid storm can present with overt heart failure and reduced ejection fraction, the presentation seen in our patient [11]. It is also important to underline that 25-43% of patients with thyroid storm may not have any identifiable precipitating factors, which was also the case for our patient [12]. Interestingly, thyrotoxicosis has been reported to cause heart failure in patients without underlying heart disease, an entity known as TCM [13]. Caused by a complex interplay of multiple pathophysiologies, TCM is most commonly seen in the setting of a longstanding hyperdynamic state in which volume expansion, increased resting heart rate, enhanced LV contractility, and reduced systemic vascular resistance collectively result in an increase in cardiac output by as much as 300% before the eventual development of high-output heart failure, with mechanisms such as increased expression of myocardial sarcoplasmic reticulum calcium-dependent adenosine triphosphatase and decreased expression of phospholamban likely playing a role [14]. Frequently coexisting with TCM is AF, another thyrotoxic complication that can contribute to the severity of symptomatic TCM via RVR and loss of atrial contribution, resulting in the compromise of LV filling and, hence, diminished cardiac output, the presentation seen in our patient. Unlike many causes of heart failure, TCM responds well to rate control, restoration of sinus rhythm, or reversion to euthyroidism, with a plausible explanation being the uncontrolled tachyarrhythmias as the primary driver for symptoms. The cardiac remodeling seen in TCM is also frequently reversible, as evidenced by the restoration of normal LV function after attaining euthyroidism in patients who initially developed a reduced ejection fraction of <50% [11]. This was the case for our patient who initially had an ejection fraction of 20% before improving to 40% after becoming euthyroid (Figure 3 and Figure 4).

Due to the life-threatening nature and substantial mortality rate that can range from 8% to 25%, particularly when the diagnosis is delayed, recognizing thyroid storm and distinguishing it from uncomplicated thyrotoxicosis is essential [15,16]. To aid in this clinical endeavor, certain scoring criteria have been put into place, with the most famous being the BWPS, a scoring system introduced in 1993 based on the degree of organ dysfunction in the setting of thyrotoxicosis. In this scoring system, a score of 45 points or more is highly suggestive of thyroid storm, whereas a score below 25 makes thyroid storm unlikely, with a score of 25-44 suggestive of an impending storm [17]. Yet despite its popularity and ease of use, the BWPS is plagued by one major caveat, its low specificity, suggesting the importance of considering the entire clinical picture as opposed to applying the scoring criteria in isolation. In line with this train of thought was our approach in diagnosing thyroid storm in our patient: we considered not only the BWPS of 55 but also the biochemical presentation, such as the abnormally low TSH levels and the abnormally high free T4 levels, the clinical presentation, such as the palpitations and significant weight loss, and the cardiac manifestations, such as the AF and de novo heart failure. Once it became clear that the patient's presentation was best explained by thyroid storm, further investigation of the underlying etiology and immediate treatment of storm became the most essential next steps.

The etiologies of thyroid storm are akin to those of thyrotoxicosis, of which Graves' disease is the most common, followed by toxic multinodular goiter and toxic adenoma [18]. Clues that suggest Graves' disease include exam findings such as diffuse goiter and thyroid orbitopathy or laboratory markers such as TSI and serum TSHR antibodies. TSHR, in particular, is known to be a strong predictor for Graves' disease and has a sensitivity of 97% and a specificity of 99% [19]. Other clues for Graves' disease include risk factors such as the female sex within the ages of 30-50, a history of autoimmune or thyroid disorders, and the recreational habit of smoking [20]. Interestingly, neither any of the aforementioned exam findings nor risk factors were seen in our patient. Instead, he tested positive for TSI and TSHR, ultimately establishing Graves' disease as the etiology of his thyroid storm.

As mentioned previously, the presence of thyroid storm should prompt immediate and aggressive treatment due to its high mortality rate. The mainstay of therapy traditionally involves beta-blockers, glucocorticoids, thionamides, and iodine, with beta-blockers for symptomatic control and the three other therapeutic options for cessation of further thyroid hormone synthesis. In particular, thionamide therapy involves the use of antithyroid drugs such as propylthiouracil and methimazole to inhibit thyroid peroxidase (TPO), the chief enzyme responsible for the formation of T3 and T4. In addition to the antithyroid effects, early initiation of thionamides has also been shown to improve or even reverse heart failure, a significant benefit given the potentially catastrophic nature of this cardiovascular complication [21,22]. Likewise, beta-blocker therapy offers similar cardiac benefits and can reduce or even completely resolve cardiomyopathy, particularly when prolonged tachycardia is likely the cause, as seen in tachycardia-mediated cardiomyopathy [23]. To support the reversible nature of TCM, a study of a series of seven patients with hyperthyroidism and congestive heart failure demonstrated an increase in the mean LV ejection fraction from 28% to 55% after appropriate treatment of thyrotoxicosis, with five patients experiencing normalization of their ejection fraction and two patients experiencing improvement from severe to mild systolic dysfunction [24]. These findings were also seen in our patient, and his ejection fraction improved from 20% to 40% after four months of therapy. Finally, the definitive treatment for thyroid storm is the removal of the underlying cause, which likely represents surgery or radioactive iodine ablation therapy for our patient with Graves' disease.

## Conclusions

De novo heart failure and thyroid storm presenting as TCM is a complex and potentially fatal clinical scenario that necessitates aggressive management in addition to a high index of suspicion for recognition. Through comprehensive assessment, timely intervention, and close collaboration among specialists, the morbidity and mortality associated with this deadly duo can be mitigated and the optimal outcomes achieved. Close follow-up after initial stabilization and planning for definitive therapy will likely be integral in preventing recurrence.
